# Developmental Pathways Are Blueprints for Designing Successful Crops

**DOI:** 10.3389/fpls.2018.00745

**Published:** 2018-06-05

**Authors:** Ben Trevaskis

**Affiliations:** CSIRO Agriculture and Food, Black Mountain Science and Innovation Park, Canberra, ACT, Australia

**Keywords:** development, crop yield, genomics, selection, plant breeding

## Abstract

Genes controlling plant development have been studied in multiple plant systems. This has provided deep insights into conserved genetic pathways controlling core developmental processes including meristem identity, phase transitions, determinacy, stem elongation, and branching. These pathways control plant growth patterns and are fundamentally important to crop biology and agriculture. This review describes the conserved pathways that control plant development, using Arabidopsis as a model. Historical examples of how plant development has been altered through selection to improve crop performance are then presented. These examples, drawn from diverse crops, show how the genetic pathways controlling development have been modified to increase yield or tailor growth patterns to suit local growing environments or specialized crop management practices. Strategies to apply current progress in genomics and developmental biology to future crop improvement are then discussed within the broader context of emerging trends in plant breeding. The ways that knowledge of developmental processes and understanding of gene function can contribute to crop improvement, beyond what can be achieved by selection alone, are emphasized. These include using genome re-sequencing, mutagenesis, and gene editing to identify or generate novel variation in developmental genes. The expanding scope for comparative genomics, the possibility to engineer new developmental traits and new approaches to resolve gene–gene or gene–environment interactions are also discussed. Finally, opportunities to integrate fundamental research and crop breeding are highlighted.

## Introduction

An increasing global population requires a 70% rise in food production by 2050 to maintain food security (Tilman et al., 2011; Fischer et al., 2014). Opportunities to increase production by expanding the total area of cultivation are limited, so increases must be delivered through higher yielding crops. This review examines the pivotal relationship between pathways controlling plant development and crop yields. Arabidopsis (*Arabidopsis thaliana*) will be used as a generic model to introduce developmental programs of plants; the blueprints that determine plant size and shape. The focus will be the pathways controlling meristem identity, determinacy, phase transitions, branching and internode elongation. Historic examples that show how these conserved pathways have contributed to crop improvement will then be highlighted. Finally, strategies to harness developmental biology for future crop improvement will be discussed within the context of current trends in plant breeding.

## Shoot Meristem Identity, Phase Transitions, and Determinacy

**FIGURE 1:**
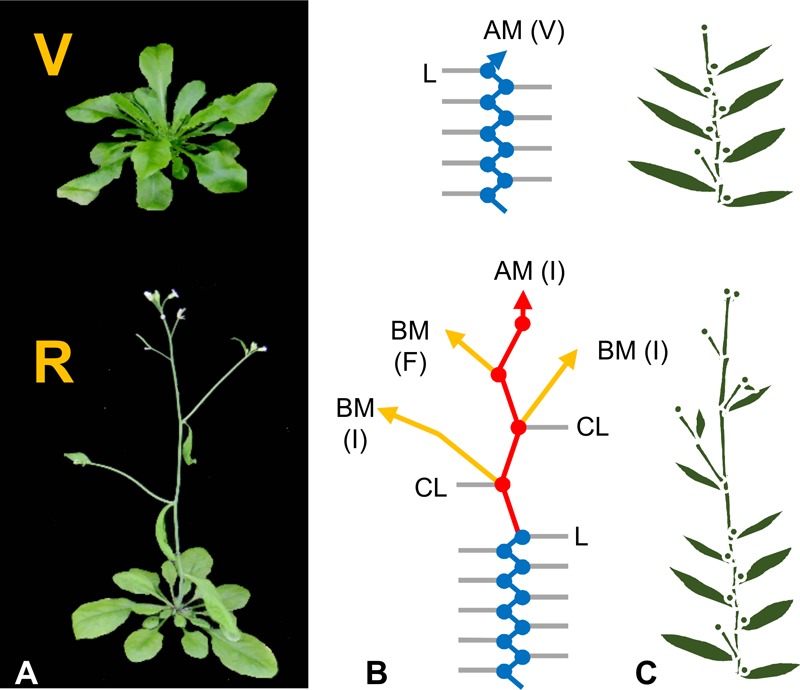
Development of *Arabidopsis thaliana*. **(A)** Arabidopsis plants in the vegetative (V) or reproductive (R) developmental phases. **(B)** Schematic representations of each growth phase. Vegetative nodes are shown as blue dots, reproductive nodes as red dots. Interconnecting lines of matching colors represent internodes. Rosette leaves (L) and cauline leaves of the inflorescence (CL) are shown as gray lines. Arrows represent shoot apices including the apical meristem (AM) and branch meristems (BM). The state of meristems are indicated in parentheses as vegetative meristem identity (V) or inflorescence meristem identity (I) for undifferentiated growing shoot tips, or as floral (F) for the lateral meristems that have differentiated into flowers. **(C)** Simplified schematic representation of plant architecture from preceding panels.

The shoot apical meristem drives above ground growth. It gives rise to structural cells, such as the cortical cells of stems, and additional meristematic zones that produce organ primordia. There are distinct phases of shoot apex development. These are easily observed during the life cycle of Arabidopsis. Arabidopsis initially grows vegetatively with the shoot apex producing leaf primordia that generate leaves arranged as a rosette (see Bowman, 1994). Reproductive development begins with a change in the morphology of the shoot apex. Inflorescence internode elongation then leads to bolting and lateral branch meristems differentiate into flowers (**Figure 1**). This developmental sequence can be divided into three phases of shoot apex meristem identity: (1) vegetative, when apices produce leaves, (2) inflorescence, when an apex contributes to inflorescence growth, and (3) floral, when an apex differentiates into a flower (**Figure 1**). Phase transitions are triggered by meristem identity genes that determine which organs develop at a shoot apex. For example, the MADS box transcription factors *APETALA1 (AP1)* and *FRUITFULL (FUL)* promote inflorescence and floral meristem identity (Mandel and Yanofsky, 1995; Ferrándiz et al., 2000). Increased *AP1* expression accelerates the transition of the primary apex to inflorescence development and promotes the production of floral organ primordia (Mandel and Yanofsky, 1995).

The primary shoot apex can grow indefinitely (indeterminate) or can terminate (determinate). The primary shoot apex of Arabidopsis normally grows indeterminately, first vegetatively, then as an inflorescence meristem that never undergoes the transition to floral development (**Figure 1**). One mechanism that terminates growth of the primary axis in some plants is the transition of the apical meristem to floral meristem identity, resulting in the production of a terminal flower. The *TERMINAL FLOWER1 (TFL1)* mutant of Arabidopsis produces a terminal flower and so exhibits determinate growth of the primary axis (Shannon and Meeks-Wagner, 1991; Bradley et al., 1997; Ohshima et al., 1997). *TFL1* encodes a poly-ethanolamine lipid binding protein (PEPB) that normally represses *AP1* expression at the shoot apical meristem (Bradley et al., 1997; Ohshima et al., 1997; Ratcliffe et al., 1999). This prevents transition of the primary shoot apex to floral meristem identity and so maintains indeterminate growth. *TFL1* mutants demonstrate that genes regulating meristem identity also control shoot determinacy.

*AP1/FUL*-like MADS box genes have been identified in the major dicot lineages and also monocots (Huijser et al., 1992; Ferrándiz et al., 2000; Benlloch et al., 2006; Kobayashi et al., 2012; Yuste-Lisbona et al., 2016). These genes have divergent roles in organ specification but have conserved roles in promoting inflorescence meristem identity and can accelerate reproductive development (Adam et al., 2007; Jaudal et al., 2015; Li Q. et al., 2016). Conserved *TFL*-like genes that repress floral meristem identity are present in diverse dicot plants and monocots (Pnueli et al., 1998; Nakagawa et al., 2002; Koskela et al., 2012). The conserved roles of *AP1* and *TFL* genes in regulating meristem identity in diverse angiosperms highlights the potential relevance of these genes to the biology of diverse crop species (**Table 1**). Specific examples from tomato (*Lycopersicon esculentum*) and bread wheat (*Triticum aestivum*) will be presented later to illustrate how the conserved meristem identity functions of these genes have been utilized in crop improvement.

**Table 1:** Overview of conservation of gene and protein function from *Arabidopsis* to crops.

Gene	Protein biochemical function	Biological role in *Arabidopsis*	Biological roles in crops	Reference
*TERMINAL FLOWER1*	PEBP signaling protein, binds receptor at shoot apex.	Maintains indeterminate growth.	A conserved role in maintaining indeterminate growth (e.g., tomato). Recruited to a role in day-length induced flowering pear (*Pyrus pyrifolia*), day-length and vernalization responses in strawberry (*Fragaria* sp.).	Iwata et al., 2012; Koskela et al., 2012; Bai et al., 2017; Koskela et al., 2017
*APETALA1, FRUITFULL*	MADS box transcription factors, activate genes during reproductive development.	Promotes transition to flowering, downstream of day-length pathway, organ specification roles (not discussed here).	A conserved role in promoting flowering in inductive daylengths. Recruited into the vernalization response of cereals.	Mandel and Yanofsky, 1995; Teper-Bamnolker and Samach, 2005; Trevaskis et al., 2007
*DELLA*	Represses growth in a gibberellin-dependent manner.	Gibberellin dependent elongation.	Gibberellin dependent elongation.	Peng et al., 1999; Dill et al., 2001; Muangprom et al., 2005
*BRANCHED1*	Represses branch meristem development and outgrowth.	Represses branch formation and outgrowth.	Represses branch formation and outgrowth.	Doebley et al., 1997; Aguilar-Martínez et al., 2007
*FLOWERING LOCUS T*	PEBP signaling protein, binds receptor at shoot apex to promote flowering.	Day-length induced florigen.	Activates flowering in inductive daylengths. Day-neutral antagonist of *TFL1* (*SP*) in tomato. Major domestication gene in sunflower (*Helianthus annuus*). Specialized roles in tuber and bulb formation in potato (*Solanum tuberosum*) and onion (*Allium cepa*).	Kardailsky et al., 1999; Kobayashi et al., 1999; Kojima et al., 2002; Turner et al., 2005; Blackman et al., 2010; Navarro et al., 2011; Lee et al., 2013

*PEBP, Poly-Ethanolamine Binding Proteins; TFL, TERMINAL FLOWER; SP, SELF PRUNING.*

## Branching and Internode Elongation

The primary axis can branch through the formation and outgrowth of secondary meristems in the axils of leaves at nodes. These “axillary buds” give rise to lateral branches that can follow similar developmental patterns to the primary axis, including the potential to give rise to additional branches. The extent of branching has a large impact on final plant size and form. Arabidopsis produces basal branches at nodes during the vegetative growth phase and branches can also develop at inflorescence nodes during reproductive growth (**Figure 1**) (Grbić and Bleecker, 2000; Long and Barton, 2000).

Apical dominance, where the shoot apex of the primary axis inhibits outgrowth of axillary buds, is a major factor influencing branching. The classical model for apical dominance is that the hormone auxin is produced at the shoot apical meristem and transported to axillary buds where it inhibits branch outgrowth (see Teichmann and Muhr, 2015). A second plant hormone, strigolactone, also inhibits lateral bud outgrowth (Gomez-Roldan et al., 2008), whereas cytokinins promote branching (Turnbull et al., 1997; Tanaka et al., 2006). Removal of the primary shoot apex (decapitation) leads to decreased auxin and increased cytokinin levels, promoting branching. Experiments with pea (*Pisum sativum*) show that decapitation also leads to increased sucrose in axillary buds (Mason et al., 2014). Increased sucrose levels trigger bud outgrowth irrespective of auxin status, which instead determines which branches continue to grow after the initial release (Mason et al., 2014). Genes that act downstream of hormone and sugar signals to control branching have been identified including the *BRANCHED1 (BRC1)* gene of Arabidopsis. *BRC1* encodes a transcription factor that inhibits both the formation of axillary meristems and bud outgrowth (Aguilar-Martínez et al., 2007).

Internode elongation influences stem and branch length (**Figure 1**). Elongation is driven by expansion of cells produced by the shoot apical meristem and by production of additional cells above nodes (at intercalary meristems). The respective contribution of cell elongation versus cell division differs between plant species. The plant hormone gibberellic acid (GA) regulates internode elongation (see Hedden and Sponsel, 2015). Bioactive GAs promote internode elongation by triggering the breakdown of growth repressing DELLA proteins, which are named after a conserved amino acid motif (Dill et al., 2001). Other hormones also influence internode elongation, including cytokinins, brassinosteroids, and strigolactones (Chory et al., 1991; Clouse et al., 1996; Szekeres et al., 1996; Bennett and Leyser, 2014).

The pathways controlling branching and stem elongation are conserved in diverse angiosperm species and are, therefore, relevant to a wide range of crops, both dicots and monocots. Indeed, the initial characterisation of *BRC1*-type genes was conducted in maize (*Zea mays*). The ancestor of modern maize, teosinte (*Zea mays*, ssp. *parviglumus*), produces many lateral branches, whereas modern cultivars typically produce a single main stem allowing high-density cultivation and increased crop yields per unit area (see Doebley et al., 1997). The reduced branching of modern maize is due to increased expression of *TEOSINTE BRANCHED1 (TB1)*, the maize equivalent of Arabidopsis *BRC1* and this is a classic example of a developmental gene that facilitated domestication of a crop (Doebley et al., 1997). Increased expression of *TB1* is associated with the insertion of a retrotransposon near the *TB1* gene (Studer et al., 2011).

## Developmental Plasticity and Phenology

Developmental plasticity allows plants to adjust growth patterns to suit different conditions. For example, the “shade avoidance response” causes stem elongation and decreased branching when plants are shaded by competitors (reviewed by Roig-Villanova and Martínez-García, 2016). These are useful developmental responses for a plant growing in a crowded canopy. Phytochrome light receptors (Phytochrome B) are central to the perception of reduced light intensity/quality and so have a major influence on the way plants channel developmental plasticity in branching and stem elongation to suit growing conditions (Franklin and Whitelam, 2005; Finlayson et al., 2010).

Phenology, the seasonal timing of life cycle events, is a particularly important example of developmental plasticity. The basis for phenology is developmental responses to seasonal temperature and light cues. These include vernalization, the promotion of flowering by prolonged winter cold (see Chouard, 1960), and day-length induced flowering (see Song et al., 2015). Vernalization and day-length flowering responses occur in many plants and regulate the timing of developmental phase transitions to coordinate critical stages of the plant life cycle, such as flowering and seed production, with optimal seasonal conditions. The precise nature of developmental responses to seasonal cues can differ across species. Developmental transitions can be triggered by short or long days, or by increasing or decreasing day-length, for example. Moreover, inductive day-lengths can promote the initiation of reproductive development in some plants, whereas the duration of subsequent developmental stages is day-length sensitive in others.

Vernalization promotes flowering of Arabidopsis (see Amasino, 2010). The vernalization response of Arabidopsis is mediated by the *FLOWERING LOCUS C* gene *(FLC)*, which encodes a MADS box transcription factor that is expressed at high levels before winter and blocks the transition to reproductive development (Michaels and Amasino, 1999; Sheldon et al., 1999). Vernalization down-regulates *FLC* and so promotes the transition to reproductive development and flowering (Michaels and Amasino, 1999; Sheldon et al., 1999). In cold climates, the vernalization requirement is a useful adaptation to prevent flowering before winter, but in warmer regions rapid flowering irrespective of vernalization can be advantageous. The molecular basis for variation in vernalization-requirement between Arabidopsis ecotypes is different levels of *FLC* activity, generally due to mutations in the *FRIGIDA* gene (Johanson et al., 2000).

Flowering of Arabidopsis is also accelerated by long days. This day-length flowering response is mediated by the *CONSTANS (CO)* gene (Putterill et al., 1995). Transcription of *CO* is regulated by the circadian oscillator and peaks in the afternoon (Suárez-López et al., 2001). When days are long this expression peak coincides with light, which stabilizes the CO protein (Valverde et al., 2004). CO then activates transcription of *FLOWERING LOCUS T (FT)* in the leaves (Suárez-López et al., 2001; Valverde et al., 2004). *FT* encodes a PEBP that is transported from leaves to the shoot apex where it promotes the transition of the shoot meristem to reproductive development by activating expression of *AP1* and other genes that promote inflorescence and/or floral meristem identity (Kardailsky et al., 1999; Kobayashi et al., 1999; Abe et al., 2005; Wigge et al., 2005; Corbesier et al., 2007; Tamaki et al., 2007). FT can be described as a florigen, which is a mobile signal that triggers flowering (see Zeevaart, 2006). Transcription of *FT* is repressed by *FLC*, so the long-day flowering response is suppressed prior to winter (Helliwell et al., 2006).

The mechanisms underlying photoperiod sensing, including the circadian oscillator and phytochrome light perception, seem to be broadly conserved through angiosperm lineages, though the functions of individual genes can diverge (e.g., Chen et al., 2014). Broad functional conservation, with potential for divergence in the roles of individual genes, also applies to the “*CO*-*FT”* day-length response pathway. For example, the rice *CO*-like gene *HEADING DATE1 (HD1)* gene of rice *(Oryza sativa)* plays an important role in regulating photoperiodic induction of *FT*-like genes but is unlikely to be a direct equivalent of the *CO* gene of Arabidopsis (Yano et al., 2000). A role for *FT*-like genes in promoting flowering is also strongly conserved, although the *FT* gene family has radiated independently in some lineages so the precise *FT*-like genes that promote flowering in a plant of interest cannot always be related to a direct ortholog in Arabidopsis (Higgins et al., 2010). There are also examples of genes from the *CO*-*FT* pathway regulating other aspects of developmental responses to photoperiod in some crops in addition to flowering, such as day-length induced storage organ development in potato and onion (Navarro et al., 2011; Lee et al., 2013). The vernalization response is an exception to the pattern of overall conservation of genetic pathways and molecular mechanisms; whereas *FLC* controls the vernalization response of Arabidopsis other genes play key roles in controlling vernalization-induced flowering outside the *Brassicaceae* (Trevaskis et al., 2003; Yan et al., 2003; Pin et al., 2012; Dally et al., 2014). Specific examples that highlight the importance of photoperiod and vernalization response pathways to crop performance are presented in subsequent sections.

## Interactions Between Developmental Processes

Plasticity in one development process influences the outcome of others. Variation in phenology, in particular, impacts many other aspects of development by fundamentally altering the plant life cycle. For example, a plant that has a longer vegetative phase produces more vegetative nodes, where leaves and branches can develop, and can accumulate more biomass. Phase transitions also influence other aspects of plant biology, including disease and stress tolerances, such as winter hardiness (Dhillon et al., 2010). As a consequence, variation in phenology, arising from environmental or genetic variation, influences other aspects of plant biology beyond simply shifting the timing of flowering. A holistic view is that variation in phenology alters the entire plant life cycle strategy and so is fundamental to adaptation to different environments.

Molecular analyses have identified points where the pathways controlling different aspects of development intersect. Protein-protein interactions are one mechanism that connects different pathways. TFL1 and FT, both PEBP proteins, act antagonistically to each other through competition for similar co-factors (Ho and Weigel, 2014). This “florigen versus anti-florigen” competition balances the promotion of flowering with maintenance of indeterminacy of the primary growth axis (see Lifschitz et al., 2014). BRC1 can bind FT to block premature floral transition of axillary meristems and so allows growth of new branches to continue irrespective of the flowering status of existing branches (Niwa et al., 2013). DELLA proteins interact with multiple regulators of phase transitions, including CO and FLC, suggesting strong interactions between gibberellin signaling and flowering pathways (Li M. et al., 2016; Xu F. et al., 2016). Other interactions between developmental processes occur through transcriptional cross-regulation. AP1, for example, represses *TFL1* by binding to the 3’ end of this gene and also regulates genes controlling GA biosynthesis (Kaufmann et al., 2010). Indeed, AP1 binds to hundreds of sites throughout the genome, as does FLC, suggesting strong potential for transcriptional cross-regulation to interconnect developmental pathways and thereby coordinate development with other biological processes (Kaufmann et al., 2010; Deng et al., 2011).

## The Importance of Developmental Biology to Crop Improvement

**FIGURE 2:**
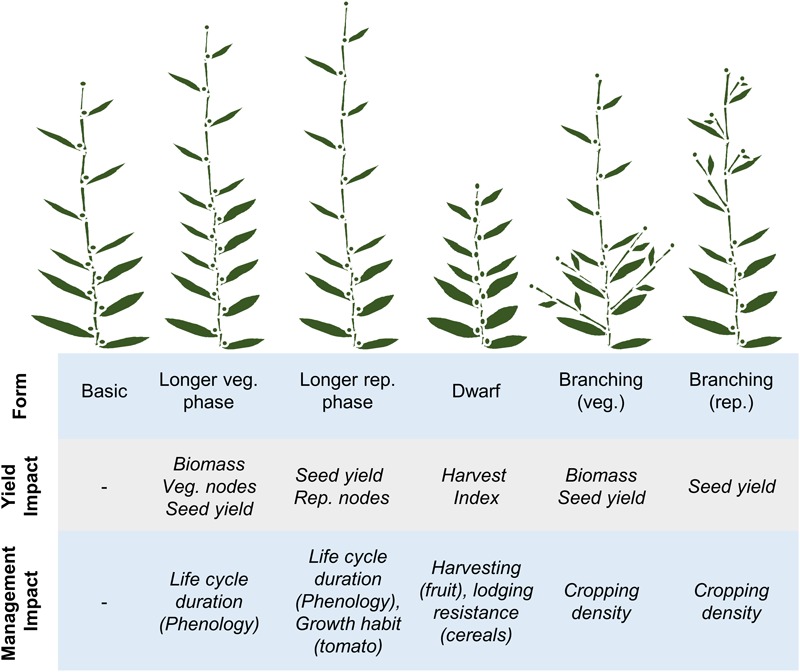
Relationships between development, components of yield and crop management practices. A basic developmental plan (far left) is contrasted with lengthened vegetative (veg.) or reproductive (rep.) phases of development, then with dwarfing or increased branching in different phases of development (from left to right). The impacts of each of the different developmental patterns on components of yield or potential crop management practices are then highlighted, relative to the basic developmental plan.

Developmental pathways are fundamental determinants of crop yield. This point is illustrated by the harvest index concept, which states that the yield of a crop under ideal growing conditions (the yield potential) is set by the proportion of plant biomass that is converted into a harvestable product (see Hay, 1995). Accordingly, yield potential can be raised by increasing crop biomass or by increasing harvest index to convert more biomass into end-product. The genetic pathways that regulate plant development control the number of leaves, branches and flowers produced by a plant and so have a strong influence on both biomass and harvest index. Consequently developmental pathways are major targets for strategies that aim to increase yield potential. Additionally developmental pathways can be used to alter plant form, or life cycle duration, to suit different management practices (summarized in **Figure 2**). Examples that highlight how developmental genes have been used to reprogram crop growth patterns to maximize yield potential and to suit modern cultivation practices are presented in the following sections.

## The Tomato *Selfpruning* Gene

Tomato exhibits sympodial growth (Atherton and Harris, 1986). The primary axis grows vegetatively to produce around 10 leaves, then develops into a terminal flower. Further growth is driven by the sympodial meristem, an axillary meristem at the last vegetative node, which generates a new branch that extends the primary growth axis. The sympodial branch follows the same pattern of growth as the primary meristem. Another sympodial meristem at the final vegetative (third) node of each sympodial branch allows the pattern to repeat. Sympodial growth can continue indefinitely and this allowed the ancestors of modern tomato to grow as vines. This growth pattern is retained in truss tomatoes.

Recessive alleles of the *SP* gene disrupt sympodial growth such that each successive sympodial unit transitions more rapidly to floral meristem identity, producing progressively fewer leaves until sympodial growth ceases (MacArthur, 1932; Silvy, 1974). This changes the growth habit of tomato from a vine to a compact bush that has more synchronous production and ripening of fruit. Recessive alleles of *SP* generate a crop that is well suited to mechanized harvesting and were critical to the foundation of the field-grown tomato industry that arose with mechanization in the second half of the 20th century (see Thompson and Blank, 2000). *SP* encodes a *TERMINAL FLOWER1*-like gene that acts at the apical meristem to delay the transition to floral identity and allow sustained sympodial growth (Pnueli et al., 1998). Recessive alleles of *SP* are caused by mutations that disrupt normal protein function (Pnueli et al., 1998). Thus, mutation of a gene controlling plant development, via meristem identity, was critical to the development of the field tomato industry.

Other genes extend or decrease the duration of sympodial development in determinate *SP* loss-of-function genotypes. *SINGLEFLOWER TRUSS (SFT)* is the tomato ortholog of *FT* (Lifschitz et al., 2006). *SFT* accelerates floral development, analogous to the Arabidopsis *FT* gene (Molinero-Rosales et al., 2004; Lifschitz et al., 2006). Lowering *SFT* activity can prolong the development of each sympodial unit and allow more sympodial units to develop (Jiang et al., 2013; Park et al., 2014). This increases the number of inflorescences and so increases fruit yield (Jiang et al., 2013). Thus, the extent of sympodial development is dependent on a balance between genes that promote or repress floral meristem identity (florigen versus anti-florigen) (Lifschitz et al., 2014). Mutations that fine tune this balance, by weakening *SFT* activity in the absence of *SP* function for example, can potentially be used to increase yield potential of field tomatoes (Park et al., 2014).

## Cereal Green Revolution Genes

Global cereal production doubled between 1960 and 1985, during what has been described as the “Green Revolution” (see Khush, 1999). This increase in productivity was achieved by a combination of improved crop management, with mechanization, irrigation and fertilizer use, and through the release of cereal cultivars that suited these new management practices. One of the key traits that adapted rice and wheat to the emergent management practices was semi-dwarf stature (see Gale et al., 1985; Dalrymple, 1986; Evans, 1993; Hedden, 2003). Semi-dwarf stature facilitated higher grain yields by preventing lodging, where fertilized crops fall over under the weight of grain (Hedden, 2003). Semi-dwarf stature also increases grain yield by changing harvest index, since reducing stem length allows a higher proportion of biomass to be allocated to grains (Syme, 1970; Walcott and Laing, 1976).

The basis for semi-dwarfism in green revolution rice is the *SEMIDWARF1* (*SD1*) gene. This gene encodes an oxidase enzyme (GA20ox-2) that is required for GA biosynthesis (Monna et al., 2002; Sasaki et al., 2002; Spielmeyer et al., 2002). Loss-of-function mutations in *SD1* lead to lowered bioactive GA levels and reduced plant height. The main driver of semi-dwarf stature in bread wheat is *REDUCED HEIGHT 1 (RHT1)*, which encodes the wheat DELLA protein (Peng et al., 1999). Semi-dwarf stature is caused by mutations that disrupt GA-induced degradation of the DELLA protein, leading to constitutive repression of internode elongation (Peng et al., 1999). The semi-dwarf genes of the green revolution highlight the importance of plant developmental pathways in setting yield potential, via determining harvest index, and further highlight how developmental pathways can tailor crops to suit different management strategies.

## Phenology, Crop Life Cycle Strategies, and Regional Adaptation

To achieve the highest possible yield, the crop life cycle must be timed to suit local field conditions. The importance of life cycle strategy is illustrated by wheat, which is grown in diverse environments through temperate and Mediterranean zones. In some regions, steady rainfall and mild seasonal temperatures allow a long growing-season, during which plants can accumulate biomass and ultimately deliver high yields. In other areas harsh winter or summer conditions limit the growing season. Genes controlling phenology allow the selection of wheat cultivars with life cycles tailored to different seasonal conditions. For example, wheats with shorter life cycles can be grown in areas with limited growing seasons and can produce grain, albeit with lower yield potential than longer season wheats.

The Australian grains industry provides a historical example of the importance of life cycle duration for regional adaption. The first wheats grown in Australia were long-season English wheats that required vernalization and long days to flower, the ancestral flowering behavior of wheats (see Evans, 1980). These wheats struggled in Australian conditions, where crops are typically sown in autumn, then grown through mild winters but must flower rapidly as temperatures increase in spring. The introduction of genes that reduce the vernalization requirement and shorten the crop life cycle was a pivotal step in developing wheats adapted to Australian conditions. This led to a massive expansion of the Australian grains industry (see Evans, 1980; Eagles et al., 2009).

The key gene controlling vernalization requirement of wheat is *VERNALIZATION1 (VRN1)*. *VRN1* promotes the transition to reproductive development and is related to *AP1* and *FUL* MADS box transcription factors of Arabidopsis (Danyluk et al., 2003; Murai et al., 2003; Trevaskis et al., 2003; Yan et al., 2003). *VRN1* is expressed at low basal levels in wheats that require vernalization to flower, but can be transcriptionally activated by prolonged cold and this promotes the transition to reproductive development (Sasani et al., 2009; Chen and Dubcovsky, 2012). Thus, it seems that the conserved capacity of *AP1/FUL*-like genes to promote flowering has been recruited to trigger vernalization-induced flowering in cereals. Wheats that flower without vernalization carry alleles of *VRN1* that are transcribed without prior exposure to cold (Danyluk et al., 2003; Trevaskis et al., 2003; Yan et al., 2003). Genotyping of historical seed samples has revealed how *VRN1* alleles that reduce vernalization requirement were introduced into Australian wheats (Eagles et al., 2009).

Variation in photoperiod requirement is another major driver of crop adaptation and genes that influence this trait have been identified in a number of crop species (e.g., Yano et al., 2000; Turner et al., 2005; Blackman et al., 2010; Hung et al., 2012; Weller et al., 2012; Xie et al., 2015). The importance of variation in photoperiod sensitivity for regional adaption is illustrated by the legume crops pea and lentil (Weller et al., 2012). The ancestral forms of these crops, which originated in the fertile crescent, flower in response to long days. Cultivation in the northern latitudes of Europe selected for cultivars that flower rapidly irrespective of daylength and complete the crop life cycle within a limited summer growing season. The genetic basis for this adaptation is loss-of-function mutations in the *EARLY FLOWERING3* gene (*ELF3*), a component of the circadian clock (Weller et al., 2012). *ELF3* loss-of-function mutations alter internal biological rhythms which activates expression of *FT*-like genes and bypasses the normal requirement for long days to trigger flowering (Weller et al., 2012).

*ELF3* loss-of-function mutations are also the basis for fast cycling barley varieties that were produced by mutation-breeding for short summer growing-seasons in the Nordic zone (Faure et al., 2012; Zakhrabekova et al., 2012). The selection of *ELF3* loss-of-function mutants in both legumes and barley highlights an emerging theme that genes encoding components of the circadian oscillator, or circadian clock, are a common basis for variation in the photoperiod sensitivity of crop plants. This includes both short and long-day flowering plants (**Table 2**). This shows that the model for daylength sensing developed in Arabidopsis, that circadian clock regulation of *CO* allows daylength specific activation of *FT*, is broadly relevant to crop species. There is likely to be more to this story, however. There are other daylength-induced floral promoters in some species, gibberellins in cereals for example (Boden et al., 2014). Alternatively, in woodland strawberry (*Fragaria vesca*) a *TFL*-like gene acts as a daylength specific repressor of flowering (Iwata et al., 2012; Koskela et al., 2012; Nakano et al., 2015; Rantanen et al., 2015). Interestingly, the circadian oscillator of barley has day-length specific states that adjust rapidly to shifting photoperiods (Deng et al., 2015b), raising the possibility that the shifting state of the circadian oscillator itself might trigger photoperiod responses, possibly through multiple downstream mechanisms.

**Table 2:** Circadian oscillator genes that influence crop development.

Clock Gene	Crop	Traits	Reference
*PSEUDORESPONSE-REGULATOR37 (PRR37/(PPD1)*	Wheat, barley, sorghum, rice, beet.	Photoperiod requirement, duration of inflorescence development, grain yield, biomass, plant height. Vernalization requirement (bolting of beet).	Turner et al., 2005; Murphy et al., 2011; Pin et al., 2012; Koo et al., 2013
*EARLY FLOWERING 3 (ELF3)*	Wheat, barley, lentil, chickpea, pea, soybean.	Photoperiod requirement, duration of inflorescence development, grain yield, biomass, plant height, leaf greenness.	Faure et al., 2012; Zakhrabekova et al., 2012; Zikhali et al., 2016; Lu et al., 2017; Ridge et al., 2017
*GIGANTEA (GI)*	Canola.	Natural variation in biological rhythms, flowering time.	Xie et al., 2015
*LUX, PHYTOCLOCK1*	Wheat, barley.	Photoperiod requirement, duration of inflorescence development.	Mizuno et al., 2012; Campoli et al., 2013; Gawroński et al., 2014
*EMPFINDLICHER IM DUNKELROTEN LICHT 1^∗^ (EID1)EID1*	Tomato.	Natural variation in biological rhythms, adaptation of biological rhythms to long days.	Müller et al., 2016

*^∗^German for more sensitive to far-red light. PPD1, PHOTOPERIOD1.*

## How Can Developmental Biology Contribute to Future Crop Improvement?

The examples above show how genes controlling development were selected to improve crop performance. This required no understanding of underlying mechanisms since developmental traits were easily observed and selected by farmers or breeders. With increasing understanding of the molecular mechanisms underlying crop developmental traits a key question is: can plant developmental biology contribute to crop improvement and future food security? Or, more specifically, can gene-level understanding of developmental biology enhance crop improvement beyond what can be achieved through traditional phenotypic selection?

A simple approach to apply developmental genetics to crop improvement is by using “perfect markers” that target sequence polymorphisms that alter gene function and directly control target traits. Perfect markers can enhance selection beyond traditional phenotype-based methods by informing parent choice, increasing the precision and scale of progeny selection, or by accelerating the rate of selection cycles. A good example of where perfect markers might out perform traditional selection is peach (*Prunus persica*) breeding. There are three dwarfing genes in peach, *DWARF* (*dw, dw2*, and *dw3*) (Hollender et al., 2016). The *dw* gene is linked to loss-of-function mutation in a gene encoding a GA receptor (Hollender et al., 2016). Similarly a candidate gene for columnar growth (vertical, non-weeping growth habit), “*BROOMY*” (*br*), has been identified (Dardick et al., 2013). Identification of the genes controlling plant height and branch angle allows perfect markers to be used for selection at the seedling stage, before plants are grown in orchards for further phenotyping. This can have a large impact on the scale and throughput of selection.

There are alternative marker-assisted selection approaches that do not require detailed knowledge of developmental biology. The use of high-density Single Nucleotide Polymorphism (SNP) marker arrays is becoming routine in crop breeding programs. SNP markers can be used as anonymous indicators of performance and developmental traits can be tracked and selected by linked SNPs or multi-SNP haplotypes (Meuwissen et al., 2001). This does not require knowledge of causal genes and can be more economical than using gene specific markers, since generic high-density marker platforms can be applied uniformly to select multiple traits. So, given the potential to apply anonymous markers to select developmental traits, the value of identifying causal genes underlying developmental variation requires deeper consideration beyond the benefits of marker-assisted selection. The following sections outline a number of ways that identifying causal genes and building detailed knowledge of gene function can contribute over and above what can be achieved by focusing on anonymous markers only. These include: (1) harnessing diversity, (2) reverse genetics, (3) re-engineering developmental traits or (4) devising new ones, and (5) using knowledge of developmental pathways to accelerate crop breeding.

## Harnessing Diversity

**FIGURE 3:**
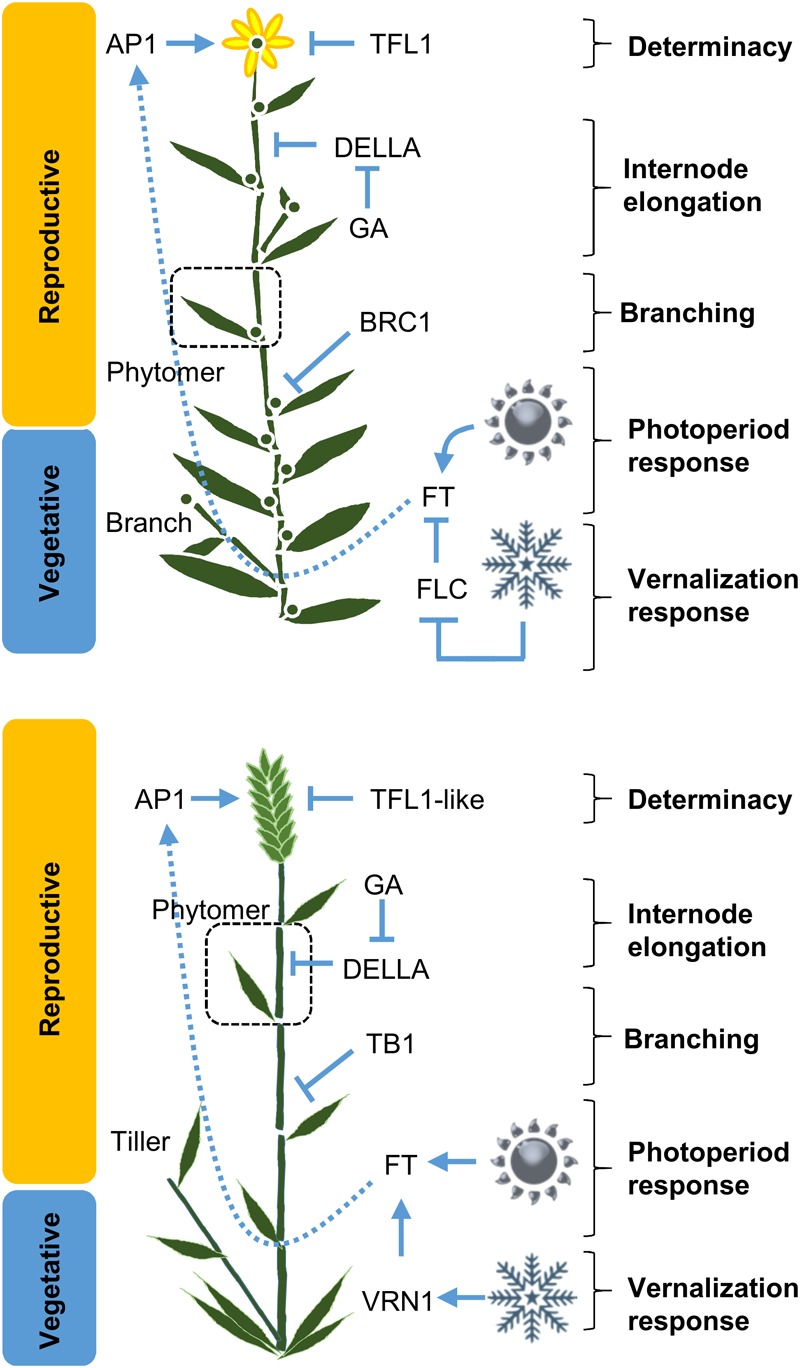
The major pathways controlling crop development. Simplified schematics of plant architecture, a dicot (top, based on Arabidopsis) versus a monocot (below, based on barley), are shown with key regulators of core different developmental processes indicated. In dicots these include; meristem identity regulated by *APETALA1 (AP1)*; determinacy regulated by *TERMINAL FLOWER1 (TFL1)*; internode elongation via gibberellin (GA) and DELLA proteins; photoperiod-induced flowering mediated *FLOWERING LOCUST (FT)*; branching *BRANCHED1 (BRC1)*. Similar genes control the equivalent processes in monocots but a key point of difference is that the vernalization response is controlled by different genes in Arabidopsis versus cereals; *FLOWERING LOCUS C (FLC)* and *VERNALIZATION1 (VRN1)*, respectively. The phytomer unit is shown (dotted line box).

Breeding for yield using anonymous markers will approach a plateau as existing variation becomes fixed in breeding programs. One way to achieve further advances is through harnessing novel diversity, from landraces or wild relatives (see Gressel, 2008; Bevan et al., 2017). For many crops there are large numbers of diverse accessions available in genetic resource centers. Useful genetic diversity can be identified and channeled into crop breeding programs by applying comparative genomics to target sequence diversity in conserved developmental regulators, such as *FT*-like genes (**Figure 3**) (e.g., Cuesta-Marcos et al., 2010; Langewisch et al., 2014). As the numbers of crop genome reference sequences increases a likely future scenario is that diversity will be targeted by breeders *in silico*, by screening for diversity in genes-of-interest in databases of re-sequenced genomes (e.g. The 3,000 Rice Genomes Project, 2014).

Diversity collections can also be used as a platform to identify diversity in crop developmental genes via Genome-Wide Association Studies (GWAS). This has proven to be an effective strategy for mapping genes controlling developmental traits in important crops including maize, rice, barley, and wheat (e.g., Tian et al., 2011; Houston et al., 2013; Edae et al., 2014; Liu et al., 2016). GWAS can provide a comprehensive overview of developmental variation in a crop. The resulting genetic information, including candidate gene sequences or linked markers, can then facilitate introgression of novel diversity into crop breeding programs. GWAS is a useful alternative to comparative genomics since it can identify novel developmental variation that is unique to crop of interest. This is important since some aspects of development have diverged in specific plant lineages (see **Table 1** and **Figure 3**). Additionally, there is scope for novel mutations (or genes) to influence the activity of conserved developmental pathways when comparisons are made across diverse plants, since factors like genetic redundancy can vary between species.

Understanding the genetic pathways controlling plant development can guide the deployment of natural diversity in crop breeding. Where there is diversity for multiple genes controlling a trait there is potential to devise strategies to stack genes to achieve larger effects, for example. Identifying multiple components in a single pathway might also identify alternative genes for a trait, with different modes of inheritance (recessive versus dominant) or different linkage relationships with other traits. Moreover, different genes in a developmental pathway can have different trade-offs with other aspects of crop performance. For example, there is potential to identify cereal clock gene mutations that influence broad aspects of daily rhythms, such as *ELF3* loss-of-function mutations, versus mutations that influence more specific clock outputs, such as recessive alleles of the *PHOTOPERIOD1* (a *PRR73* gene) that alter daylength responses with less impact on overall clock function (Campoli et al., 2012; Zakhrabekova et al., 2012).

An interesting example of where comparative genomics, GWAS and knowledge of genetic pathways are converging to offer new ways to harness crop diversity comes from genes controlling inflorescence (panicle or spike) architecture in cereals. These genes determine the extent to which lateral branch meristems develop at nodes of the inflorescence. For example, low transcript levels of the wheat *FT1* gene slow meristem phase-transitions in the spike and enhance the formation of paired spikelets, where two spikelets form at inflorescence nodes instead of the normal single spikelet (Boden et al., 2015). Other genes, including the *TB1* gene of barley and wheat (*HvTB1, TaTB1)*, suppress branch outgrowth from inflorescence nodes (Ramsay et al., 2011; Dixon et al., 2018). An AP2/ERF transcription factor (**Table 3**) has a conserved role in suppressing inflorescence branching in maize, rice, barley, and wheat, and loss-of-function mutations in this transcription factor generate highly branched inflorescences in each of these cereals (Chuck et al., 2002; Komatsu et al., 2003; Zhu et al., 2003; Dobrovolskaya et al., 2015; Poursarebani et al., 2015). Inflorescence branch number influences grain number per plant. Access to diversity in multiple genes controlling inflorescence architecture will allow the potential value of new inflorescence branching patterns to be explored in the major cereal crops. There are also opportunities to explore the regulation of cereal inflorescence branching by hormones and sugars, based on knowledge developed in Arabidopsis (e.g., Mason et al., 2014).

**Table 3:** Regulators of cereal inflorescence structure.

Protein Description	Maize	Barley	Wheat	Rice	Reference
C2H2 Transcription factor	*RAMOSA1 (RA1)*	N	N	N	Vollbrecht et al., 2005.
Lateral Organ Boundary Domain	*RAMOSA2 (RA2)*	*VRS4 (HvRA2)*	N	N	Bortiri et al., 2006; Koppolu et al., 2013.
Trehalose-6-phosphatase	*RAMOSA3 (RA3)*	*SISTER OF RAMOSA 3 (HvSRA3)*	N	N	Satoh-Nagasawa et al., 2006.
TCP transcription factor (BRC1)	*TEOSINTE BRANCHED1 (TB1)*	*HvTB1*	*TaTB1*	*FINECULM/ OsTB1*	Doebley et al., 1997; Takeda et al., 2003; Ramsay et al., 2011; Dixon et al., 2018.
Homeodomain transcription factor	*GRASSY TILLERS*	*VRS1*	N	N	Komatsuda et al., 2007; Whipple et al., 2011.
APETALA2-like gene family	*INDETERMINATE SPIKELET1 (IDS1), SISTER OF IDS1*	*ZEOCRITON*	*Q*	*SUPER-NUMERARY BRACT, Oryza sativa OsIDS1*	Lee et al., 2007; Chuck et al., 2008; Lee and An, 2012; Houston et al., 2013; Greenwood et al., 2017.
AP2/Ethylene Response Factor	*BRANCHED SILKLESS*	*COMPOSITUM2*	*BRANCHED HEAD MULTIROW SPIKE*	*FRIZZY PANICLE, BRANCHED FLORETLESS*	Chuck et al., 2002; Komatsu et al., 2003; Zhu et al., 2003; Dobrovolskaya et al., 2015; Poursarebani et al., 2015.
Short-internodes1-like	N	*VRS2*	N	N	Youssef et al., 2017.
Florigen (poly-ethanolamine binding protein)	*Zea mays CENTRORADIALIS8 (ZCN8)*	*FLOWERING LOCUS T-like 1 (FT1)*	*FT1*	*HD3A/RFT1*	Turner et al., 2005; Meng et al., 2011; Boden et al., 2015.
Topless co-repressor (TPL)	*RAMOSA ENHANCER LOCUS2*	N	*TaTPL*	*ABERRANT SPIKELET AND PANICLE1*	Gallavotti et al., 2010; Yoshida et al., 2012; Liu et al., 2017, 2018.
Histone Demethylase	N	*VRS3*	N	N	Bull et al., 2017; van Esse et al., 2017.

*N indicates component not identified/characterized. Notes on abbreviations: C2H2 refers to a class of zinc finger transcription factor. TCP1 is named after the proteins TEOSINTE BRANCHED1 (TB1) from maize (*Zea mays*), CYCLOIDEA (CYC) from snapdragon (*Antirrhinum majus*), and PROLIFERATING CELL NUCLEAR ANTIGEN FACTOR1 from rice (*Oryza sativa*). Other undefined abbreviations include *VRS*: row type gene and *HD3A*: *HEADING DATE 3A*. *Q* is simply the *Q* gene, not an abbreviation.*

## Reverse Genetics

There is potential to move beyond the scope of natural variation by applying reverse genetics. Before considering potential applications of reverse genetics to the developmental biology of crops, it is important to consider the genetic architecture of developmental traits. So far the main focus of this review has been simple developmental traits that are controlled by limited numbers of genes. These traits are ideal targets for reverse genetics since altering the activity of a single gene can trigger large phenotypic changes. More complex developmental traits are also important but are less attractive as initial targets for reverse genetics. These will be discussed later. Another important consideration is the nature of functional variation that give rise to developmental traits. Historical examples include loss or gain-of-function mutations, of varying strengths (**Table 4**). These highlight a number of molecular mechanisms that can be targeted by reverse genetics to engineer crop development. Three examples of ways that reverse genetics can be applied to crop developmental biology are discussed below.

**Table 4:** Examples of molecular mechanisms underlying developmental variation in crops.

Crop	Gene	Mutation	Molecular Outcome	Trait Outcome	Reference
Rice	*SD1*	Nucleotide polymorphism, amino acid substitution.	Loss of enzyme activity, decreased gibberellins.	Reduced plant height, higher harvest index.	Monna et al., 2002; Sasaki et al., 2002; Spielmeyer et al., 2002.
Tomato	*SFT*	Nucleotide polymorphism, amino acid substitution.	Reduced protein function.	Delay of developmental transitions, higher fruit yield.	Krieger et al., 2010.
Rice	*SPL14*	Nucleotide substitution, microRNA binding site disrupted.	Increased gene expression.	Increased panicle branching, increased grain yield.	Jiao et al., 2010; Miura et al., 2010.
Wheat	*RHT1*	Deletion of part of gene causing translational frame shift.	Loss of protein domain required for gibberellin-induced breakdown.	GA-insensitive, semi-dwarf stature, higher harvest index.	Peng et al., 1999.
Barley	*VRN2*	Deletion of gene.	Loss of flowering repressor.	Early flowering without vernalization.	Yan et al., 2004.
Maize	*TB1*	Transposon insertion.	Elevated expression.	Repression of tillering, allows high-density sowing.	Studer et al., 2011.

*SD1, SEMIDWARF 1; SFT,Solanum FLOWERING LOCUS T; SPL14, SQUAMOSA PROMOTER BINDING PROTEIN-like 14; RHT1, REDUCED HEIGHT 1; VRN2, VERNALIZATION 2; TB1, TEOSINTE BRANCED1; GA, gibberellic acids.*

An exciting area for reverse genetics in crops is the potential to engineer loss-of-function traits in polyploid crops. Bread wheat, for example, is an allohexaploid that has three diploid equivalent genomes (A, B, and D). For most wheat genes there is redundancy between the three genomes, so loss-of-function mutations are unlikely to impact phenotype and be selected by traditional breeding methods. For example, loss-of-function mutations in gibberellin biosynthesis genes that give rise to semi-dwarf stature would have been difficult to deploy in wheat. Access to genome sequences, combined with “TILLING (Targeting Induced Local Lesions IN Genomes) by sequencing” allows efficient identification of loss-of-function mutations on all three wheat genomes, which can then be stacked in single line using corresponding molecular markers (Krasileva et al., 2017). This approach allows recessive “loss-of-function traits” that have been deployed in diploid cereals to be translated to hexaploid wheat, where recessive traits are less likely to have arisen historically due to genetic redundancy. Already there are examples of developmental traits known from diploid cereals that have been selected in bread wheat (Kippes et al., 2016). Similar approaches can be applied to other polyploid crops. This is an area where genome biology has the potential to transform breeding of several major crops that are polyploid (e.g., wheat, cotton, canola, sugarcane).

Reverse genetics can also be used to generate gain-of-function traits. Generating mutations in microRNA target sites is an effective way to induce genetic gain-of-function. A number of developmental genes, including members of *AP2*-like and *SQUAMOSA-PROMOTER BINDING PROTEIN*-like (*SPL*) gene families, are regulated by microRNAs that bind to target sites in mRNAs and trigger transcript cleavage (reviewed in Tang and Chu, 2017). Mutations in the microRNA binding sites present in these target genes prevents microRNA binding and mRNA cleavage, leading to up-regulation of gene activity and gain-of-function traits. For example, the mRNA of the wheat *Q* gene has a 21 bp target site for microRNA172 (miR172) (Debernardi et al., 2017; Greenwood et al., 2017). Mutations in the miR172 target site can increase *Q* mRNA levels and are likely the basis for domestication alleles that reduce inflorescence length and increase spike threshability (Debernardi et al., 2017; Greenwood et al., 2017). Mutations in the miR172 target site of a related *AP2-*like gene from barley also alter spike architecture, giving rise to cleistogamy (closed florets at anthesis) or compact spikes (Nair et al., 2010; Houston et al., 2013). Similarly, mutations in the miR156 binding site within the rice *SPL*-like gene *IDEAL PANICLE ARCHTECTURE/WEALTHY FARMERS PANICLE* can alter inflorescence architecture to increase grain yield (Jiao et al., 2010; Miura et al., 2010).

Another area of emerging interest is the potential to apply reverse genetics to generate subtle alteration of gene activity to fine tune developmental traits. For example, altering *SFT* activity in tomato can optimize developmental patterns to maximize yield (Krieger et al., 2010). TILLING the promoter of *FT*-like genes in crops could generate a graduated series of developmental patterns, which can be evaluated in field trials. Importantly, these would be single gene effects in a common genetic background, so different alleles can be compared and evaluated with precision. Producing a range of discrete activities of a single gene in a common genetic background using traditional plant breeding approaches would be challenging.

The advent of efficient gene editing offers another approach to generate novel genetic variation in crops (see Globus and Qimron, 2017; Scheben et al., 2017). Gene editing allows gene functions to be disrupted and/or novel functions to be introduced at specific points in the genome (Shan et al., 2013). Considered from the viewpoint of a single gene this is a powerful technology. Even more powerful is the potential to target multiple genes (Rodríguez-Leal et al., 2017). This can allow manipulation of multiple targets in a common genetic background to overcome genetic redundancy where function is encoded by multiple genes or by multiple copies in a polyploid crop. It also allows the activities of different pathways to be altered by targeting multiple genes simultaneously. There are potential technological and regulatory hurdles, however. Gene editing requires that genes are introduced into crop targets via transformation (stable or transient) and will potentially be subject to strict regulatory requirements that are currently applied to genetically modified crops (Globus and Qimron, 2017). Clarity with respect to whether gene edited plants will be regulated as genetically modified organisms is required before this technology can be widely applied in crops.

## New Ways to Utilize Developmental Variation in Crops

There is potential for novel applications of plant development in crop breeding. One area is hybrid vigor (heterosis). Hybrid vigor, where the progeny of an inter-cross out-performs either parent, is often applied by plant breeders to increase crop yields. If developmental patterns and plant architecture are critical to increasing plant yield then one prediction is that genes controlling plant development should contribute to hybrid vigor. There is evidence this is the case. Heterozygosity at *FT* increases yield of tomato, by optimizing development and plant architecture (Krieger et al., 2010; Jiang et al., 2013). Similarly, heterozygosity at *DAYS TO HEADING8* increases grain number of rice and heterozygosity for the MADS box gene *AGAMOUS-like 50*, which influences the timing of flowering, increases biomass of Arabidopsis hybrids (Li D. et al., 2016; Seymour et al., 2016). This raises the question of whether variation in developmental genes can be used to predict hybrid performance when different cultivars are inter-crossed. One indicator would be the extent to which developmentally regulated yield components contribute to hybrid vigor in any particular crop. For example, if increased yield is achieved through the production of more flowers then developmental genes controlling inflorescence structure are likely to be at least partly underlying heterosis. A related theme, where developmental biology could influence hybrid seed production, is fertilization-independent seed production, or apomixis. Transferring genes for apomixis to crops could be used to perpetuate hybrid vigor (Hand and Koltunow, 2014).

Another area for novel developmental traits is below ground development. Knowledge of pathways controlling root development has advanced rapidly in Arabidopsis but, with the exception of an advanced understanding of genetic control of nodule development in nitrogen-fixing legumes (e.g., Vernié et al., 2015), there are few examples that link gene function to adaptive root biology in crops. Intuitively, variation in root depth or total root volume seems likely to contribute to crop adaptation, particularly to specific soil types, and increased focus on genetic control of root development is an interesting area for further research. One challenge will be to develop phenotyping methods that allow root phenotypes to be assayed in field grown plants (Richard et al., 2015; Pineros et al., 2016; Wasson et al., 2016). In this context the high degree of plasticity of root development, responding to soil density, moisture and nutrients, will present challenges. A recent finding that the *VRN1* gene of wheat and barley influences root development, in addition to regulating reproductive development, highlights the potential for genes that influence above ground development to have pleiotropic effects on root architecture (Voss-Fels et al., 2018).

## Revisiting Old Developmental Traits

Many beneficial developmental traits, such as the reduced tillering of maize conferred by *TB1*, are already fixed in crops. So, can knowledge of the molecular basis of these traits be used to increase crop yields beyond what has already been achieved? One suggestion is that knowledge from established crops can applied through translation to related species that are still undergoing domestication. For example, known domestication genes can be recapitulated in wild/near-wild plants, either by screening for mutations or through genetic modification (see Østerberg et al., 2017). This is a valid suggestion, but the crop targets need to be considered carefully. To be useful in existing agricultural zones any new domestication efforts would need to focus on plants that can rival existing crops, either for productivity or end product value. Alternatively, new crops could be bred for cultivation in agro-ecological niches that do not suit existing crops; new crop rotation opportunities or cultivation in marginal areas, for example.

There are situations where the developmental traits/genes that were initially selected and fixed in crops might not be the optimal solution. In these instances, knowledge of the molecular basis of developmental traits could guide strategies to achieve future yield gains. One example is the potential for alternative green revolution genes for wheat. The *RHT1* alleles that have been used to breed semi-dwarf wheats have detrimental pleiotropic effects, such as reducing coleoptile length and grain size (Ellis et al., 2004). Knowledge of the genes controlling plant height allows exploration of alternative genetic solutions. Alternative alleles of *RHT1* can potentially be used to reduce negative pleiotropic effects (Chandler and Harding, 2013). There is also potential to use different genes to reduce height without detrimental impacts on other traits (Ellis et al., 2004; Ford et al., 2018). Another way to generate alternative genes for traits of interest is to apply comparative genomics and reverse genetics. For example, TILLING could be used to generate and stack recessive *SD1* mutations in wheat, to transfer the rice green revolution gene to wheat.

## Utilizing Knowledge of Development at the Genome-Wide Scale in Plant Breeding

**FIGURE 4:**
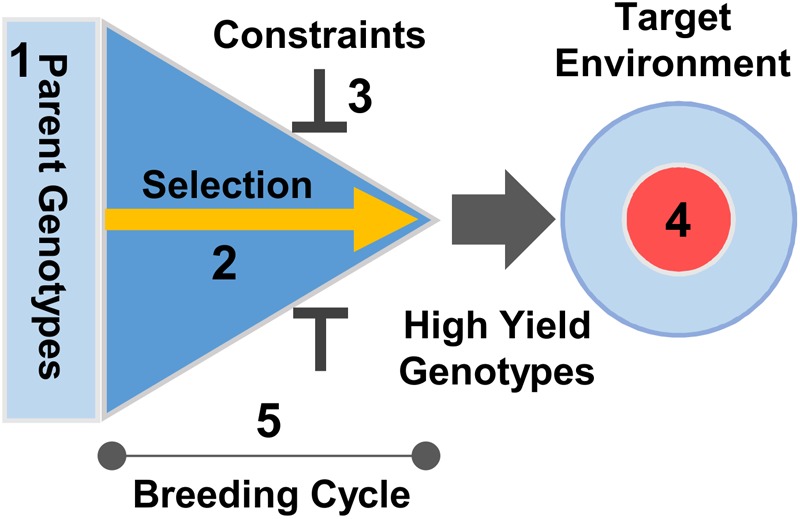
Application of development genetics to crop breeding. Developmental genetics can accelerate the rate of genetic gain for yield by influencing a number of factors in the breeding process. Selection efficiency and scale can be increased using molecular markers to inform choice of parents and to select progeny **(1, 2)**. Knowledge of trait interactions can be used to balance trade-offs between selecting for increased yield and pleiotropic impacts on other traits (e.g., quality) to ensure that increasing yield is compatible with other selection constraints **(3)**. Understanding how gene-environment interactions influence development can be used to precisely target selection for yield to suit specific growing regions or climates **(4)**, maximizing achieved yield (“on farm” yield delivered in targeted growing environments). Finally, knowledge of phenology genes can be used to develop growth conditions for speed breeding (Watson et al., 2018) or transgene tools (Flachowsky et al., 2011) to accelerate the breeding cycle **(5)**.

Modern breeding programs typically shuffle variation in large numbers of genes controlling multiple traits, spanning from yield potential, stress tolerance, disease resistance to end-use quality. One question that has not yet been addressed is whether knowledge of developmental biology can be harnessed to accelerate the rate of yield gain in the complex context of plant breeding. Knowledge of developmental gene function can influence several factors that determine the rate of genetic gain in a breeding program (**Figure 4**). Subsequent discussion will focus on three specific areas; dissecting developmental components of yield potential, resolving pleiotropy, and optimizing development to maximize potential yield for specific target environments.

**FIGURE 5:**
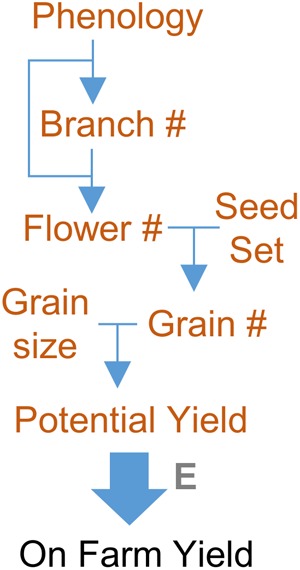
Focusing on yield component traits to resolve genetic complexity of yield. Yield potential is a complex trait, determined by numerous genes and genetic interactions. These genetic factors can be resolved by focusing on yield component traits. For a grain crop these include phenology together with branch, flower, and grain number (#). Genes controlling these yield component traits can be resolved and identified. The effects of genes controlling yield components, and the interactions between them, can be used to predict yield potential. Actual yield will be determined by how the environment (E) influences the individual yield components. Focusing on yield components, rather than total yield, can also be used to resolve the gene-environment interactions that influence on farm yield.

The genetics of yield is typically complex. Nevertheless, in many crops, overall yield can be divided into subcomponent traits. For example, seed number is an important yield component for a cereal crop (see discussion of yield components in Reynolds et al., 2012). Yield subcomponents are in turn determined by developmental traits such as phenology, branch number and flower number, as discussed above (**Figure 5**). The genetic basis of these subcomponent traits can be resolved. For example, the genetic architecture of phenology is well understood in several crops, including maize, wheat, rice, barley, legumes and canola, and there is deep knowledge of the overall molecular pathways and genes controlling specific yield subcomponents of some crops (Buckler et al., 2009; Hori et al., 2015; Xu L. et al., 2016; Li et al., 2017; Würschum et al., 2018). This knowledge can be applied in plant breeding by applying marker assisted selection to optimize individual yield components to increase overall yield potential. The precise selection approach will vary between crops depending on the genetic architecture of the target traits. Where there are major effect genes or epistasis (e.g., wheat phenology) a limited number of molecular markers can have a large impact on selecting for yield component traits, through selection of parents with compatible genotypes or through the use of marker assisted selection to screen progeny and thereby increase selection precision and throughput. Where small additive effects of large numbers of genes underlie variation for yield components (e.g., maize phenology) there is potential for genetic markers to be used in genomic selection approaches that target specific alleles of genes controlling development.

Another factor that confounds selection is pleiotropy, where selection for a particular developmental trait has flow-on effects to other traits (see **Figure 2**). For example, variation that accelerates the transition to reproductive development of wheat can lead to fewer inflorescences per plant, and thus lower grain/seed yield (see Slaefer et al., 2009). Additionally, developmental traits can also influence other traits, such as abiotic stress tolerance or end-use quality (Fowler et al., 2016). This is an important consideration since developmental traits are not the only important traits. Knowledge of genes underlying yield component traits allows pleiotropic effects to be dissected with precision using near-isogenic lines or similar genetic stocks (e.g., Steinfort et al., 2017). This knowledge can be used to refine breeding strategies, to design optimal developmental patterns that maximize yield with acceptable trade-offs with other traits. Similarly, targeted genomics approaches that have been applied to Arabidopsis research, such as genome-wide identification of transcription factor binding sites, can potentially be applied to crops to uncover and predict interactions between development genes and other traits (Deng et al., 2015a).

Site-to-site and year-to-year variation also confounds phenotypic selection for developmental traits and this presents a major challenge to breeding for many crops. Gene-environment interactions are particularly relevant to aspects of development that are regulated by temperature or light cues that vary according to location or management practices (e.g., phenology). An improved understanding of how gene-environment interactions influence crop development can allow developmental patterns to be optimized for specific target environments. One specific area that warrants further research is developmental responses to higher ambient temperature. Increasing temperatures influence crop developmental patterns, typically accelerating flowering and reducing yield (Hemming et al., 2012; Hatfield and Prueger, 2015). Current understanding of how elevated temperatures influence development of crops, and the extent of any natural variation in this response, is limited. Further research could ensure that breeders have access to the necessary genetic variation to maximize yield potential in the warmer climates of the future.

As crop breeders begin to apply genotyping-by-sequencing there will be opportunities for plant breeding to be closely linked with genome biology research. For example, training populations used in genomic selection programs can be used for gene mapping. Breeding pedigrees can also be used for gene mapping, as illustrated by autozygosity mapping in human genetics where homozygosity for allelic variation within a pedigree can be used to locate chromosomal regions carrying a genetic disease (see Carr et al., 2013). A close link between basic and applied crop biology might also allow reverse genetics to be deployed directly in breeding programs.

With sufficient understanding of crop biology it will be possible to integrate gene and environment information to predict overall developmental behavior and flow-on effects to other traits. Models that predict flowering dates for specific crops at particular sites are already used as management and research tools, such as Agricultural Production Systems sIMulator (APSIM) (Keating et al., 2003). A future aspiration should be to use genome sequences and climate data to predict crop performance (“bio-prediction”). This would have applications in crop breeding and also allow crop management strategies to be targeted to specific varieties of a crop, for specific environments. This will require integrated analysis of genomes with other types of “big data”, so there will be the need for analytical methods that can process large volumes of different data types. Machine learning might be one way to address this challenge (O’Brien et al., 2015).

## Conclusion

Developmental pathways control patterns of plant growth that determine crop yield. Historical examples highlight the transformative impact that variation in developmental genes has had on crop performance. Now, with increased understanding of conserved pathways controlling plant development and access to crop genome sequences, diversity in genes controlling development can be rapidly and efficiently channeled into crops. This is paralleled by emerging reverse-genetics technologies that offer new ways to harness developmental biology for crop improvement. Applying these advances in plant developmental biology to crop breeding will contribute to increasing crop yields to meet future demand.

## Author Contributions

The author confirms being the sole contributor of this work and approved it for publication.

## Conflict of Interest Statement

The author declares that the research was conducted in the absence of any commercial or financial relationships that could be construed as a potential conflict of interest.
